# Experimental study on frost-formation characteristics on cold surface of arched copper sample

**DOI:** 10.1371/journal.pone.0208721

**Published:** 2018-12-11

**Authors:** Tingkun Chen, Qian Cong, Jingfu Jin, Kwang-Leong Choy

**Affiliations:** 1 Key Laboratory of Bionic Engineering, Ministry of Education, Jilin University, Changchun, P. R. China; 2 State Key Laboratory of Automotive Simulation and Control, Jilin University, Changchun, P. R. China; 3 College of Biological and Agricultural Engineering, Jilin University, Changchun, P. R. China; 4 Institute for Materials Discovery, University College London, London, United Kingdom; Institute of Materials Science, GERMANY

## Abstract

The present work investigates the process of frosting formation on arched copper samples with different surface temperatures, calculated the thickness of the frost layer by using the scale method, and analyzed frost lodging, melting, and other phenomena that appeared during the frost-formation process. The results showed that the frosting process on an arched surface can be divided into ice-film formation, rapid growth of the frost layer, and stable growth of the frost layer. Meanwhile, the phenomena of frost-branch breakage, lodging, and melting were observed. The surface temperature had a large effect on the frost formation and thickness of the frost layer, e.g., the formation time of the ice film on a surface at -5°C was the longest (~135 s), the frost layer formed on a surface at -20°C was the thickest (~660 μm). When microscopic observation of the frosting process was accompanied by calculation of the frost-layer thickness, it could be seen that the appearance of the frost branches was affected by the different thermal conductivities of the frost layers, undulating surface of the ice film, and temperature difference between the layers. The changes in the frost branches and the soft surface of the frost layer also affected the growth of the frost layer. The findings of this study are expected to provide guidelines for optimization of conventional defrosting methods.

## 1. Introduction

When the surface temperature of a material is lower than the dew point, heat transfer will cause water vapor in a humid environment to condense on the exposed surface and form a layer of frost. This natural phenomenon is widespread in multiple industries such as refrigeration, air-conditioning, and aviation [1–5]. Frost accumulation on cold surfaces can lead to socioeconomic loss and affect the operational efficiency and safety of equipment, and it may even jeopardize human lives. For instance, frost accumulated on a heat pump leads to an increase in the heat-transfer resistance and a reduction in the pump’s operational efficiency [6,7]. Meanwhile, frost often accumulates on evaporators of refrigeration systems, resulting in increased energy consumption to attain the same refrigerant effect [7,8]. Moreover, the accreted frost on the cabinet of a refrigerator not only affects the refrigerating efficiency, but also deteriorates the quality of the food stored inside [9,10]. Of course, many industries and our daily lives are affected by frosting, such as frost on the front glass of cars or spectacles, which reduces visibility, and frost accumulating on aircraft wing surfaces, which compromises the aerodynamic parameters.

Many efforts have been made to mitigate the damages caused by accumulated frost, and many defrosting methods have been developed. These methods can be divided into three categories: mechanical ways to directly remove the accreted frost, such as manual de-frosting; heat-based methods to melt the covered frost layer, such as blowing hot air or electric heating of the surface; and chemical methods to delay frosting [10–12]. However, these conventional defrosting methods have been shown to have many drawbacks during engineering applications. For example, mechanical de-frosting methods require high operating costs, the amount of energy wasted by a heat-based defrosting method increases with the complexity of the system, and chemical defrosting methods are detrimental to the environment, corrode structures, and shorten the service lifetime of equipment [12–14]. Therefore, new anti-frosting and de-frosting methods must be developed to alleviate the hazards of accreted frost.

Since the discovery of the hydrophobicity of lotus leaves in the last century [15], many researchers have been inspired by the self-cleaning properties of the leaves and have committed to fabricating hydrophobic or super-hydrophobic surfaces to reduce frost formation. Therefore, the use of super-hydrophobic surfaces is regarded as the most promising anti-icing or anti-frosting method owing to their extraordinary water-repellency [12,16–18]. Sommers and co-workers [19–21] fabricated samples with different wettabilities to alter their effect on the frosting parameters, and the defrosting performance was tested on the surfaces with different wettabilities and micro-patterns. However, even though hydrophobic and super-hydrophobic surfaces are expected to retard or prevent frost formation, many reports in the literature reveal that after multiple freeze-thaw cycles under certain experimental conditions, some superhydrophobic surfaces show poor performance such as low durability, weak pollution resistance, and low adhesion force between the coating and substrate [22–27]. Meanwhile, the preparation of hydrophobic or super hydrophobic surfaces is complicated and costly, and there have yet to be reports of their successful application in engineering.

To optimize the conventional anti-frosting and defrosting methods and mitigate their drawbacks, many researcher adopted mathematical modeling to study and analyze the frosting process on the surfaces of engineering components. For example, Hermes and co-workers [28,29] proposed a semi-empirical model, combined with experimental data, for predicting frost accretion on hydrophobic and hydrophilic surfaces and for predicting the conductivity of the frost; Breque and Nemet [30] designed a model to predict frost growth on a heat exchanger; Na and Webb [31] and Piucco et al. [32] adopted phase-change kinetics to analyze the nucleation process, and they concluded that droplets should be formed more easily than frost crystals on a cold surface. Meanwhile, published studies on the frosting process mostly adopted the vertical method to observe the condensation process of water vapor on the smooth surface of a substrate, and the horizontal method was used to observe the frost-formation process. However, frost formation takes place earlier at the edge than at the center of a sample, thus disturbing the observation of the frost-formation process at the center.

Therefore, the frost-formation process at different surface temperatures was observed during the study reported here, especially the changes in frost branches during the formation process. In addition, we investigated the effects of different surface temperatures on the frosting process and the thickness of the frost layer, as well as the reasons for the changes in the frost branches. The results of this study could offer guidelines for developing new defrosting methods with fewer drawbacks, such as disturbing the frosting process or implementation of conventional anti-frosting and defrosting methods, for determining the best defrosting duration, and for reducing energy consumption and cost.

## 2. Materials and methods

### 2.1. Methods

**Fig 1:**
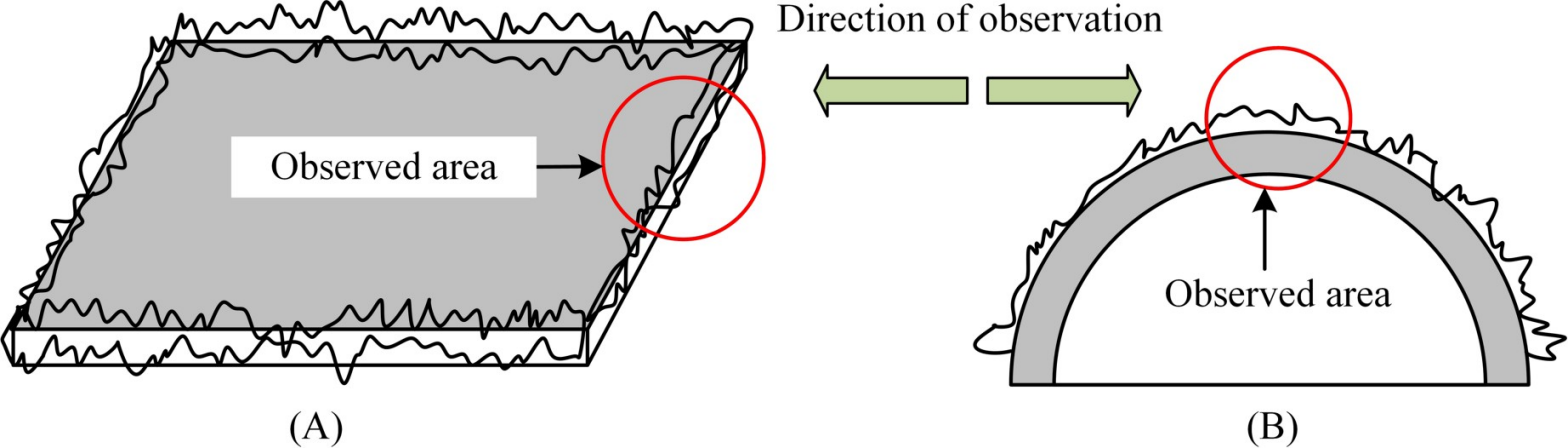
Observation field of frost formation on different surfaces. (A) Smooth sample; (B) arched sample.

As reported in the literature, frost formation is often examined on flat surfaces by using the horizontal observation method. However, water vapor first condenses around the edge of the sample, which is where frost crystals begin to appear, as shown in Fig 1A. As freezing progress over time, the frost layer located on the edge region of the sample begins to increase, thus affecting the freezing process on the central area of the cold surface (as viewed from the horizontal direction). Hence, to avoid the defects generated by the horizontal observation method and to establish a good observation field, an arch-shape sample, as shown in Fig 1B, was fabricated for the tests. In addition, the corresponding observation device was set up for the arched sample.

The frost-formation process is complex and influenced by many factors. Since the cold surface temperature, ambient temperature, and ambient humidity are the main factors of frost formation, our observation of the frosting process on the cold surface was carried out at a stable ambient temperature and under stable humidity conditions. The effect of surface temperature on the frosting characteristics was quantitatively analyzed.

### 2.2. Experimental apparatus

**Fig 2:**
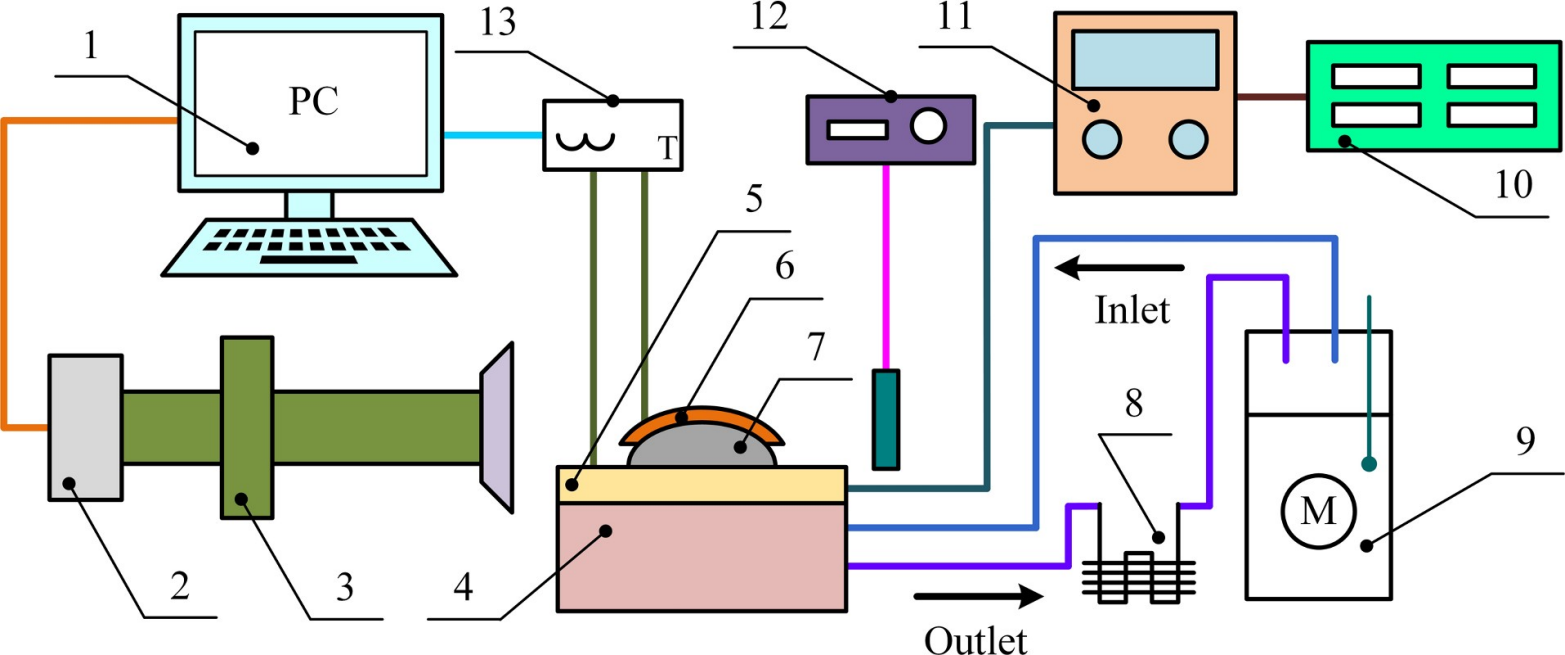
Schematic representation of the experimental setup. (1) PC; (2) charge-coupled device (CCD) camera; (3) microscope; (4) cooling stage; (5) semiconductor refrigeration unit; (6) arched copper specimen; (7) sample holder; (8) air cooled condenser; (9) water bath; (10) temperature controller; (11) DC power; (12) lighting system; (13) temperature recorder.

A microscopic set up was used to observe the frosting process on the surface of the arched sample from the horizontal direction, as shown in Fig 2. The set up consisted of a refrigeration system, thermoregulation system, cooling system, data acquisition system, and other components. The refrigeration system included a semiconductor refrigeration unit to realize the goal temperature of the sample, cooling stage, and sample holder. The thermoregulation system consisted of a temperature controller, direct-current (DC) power supply, K-type thermocouple, and it was used to synchronously control and measure the temperature during a test. The accuracy of the thermocouple was ±1.0%. In order to decrease the effect of the heat generated by the semiconductor refrigeration unit on a test, an experimental device equipped with a cooling system was used to dissipate heat. This cooling system was composed of an air-cooled condenser, water bath, and so on. The data acquisition system, which consisted of the microscope with a charge-coupled device (CCD) camera and a temperature collector, could simultaneously collect a video of the frost-formation process and record the changes in surface temperature of the sample and the temperature of the semiconductor refrigeration unit during the experiment. The experimental data were collected every one second.

During the experiments, the entire experimental setup was placed in a climate chamber. The climate chamber contained the temperature and humidity control systems, and the control accuracies were ± 0.2°C and ±2.0% relative humidity (RH), respectively.

### 2.3. Materials

Copper is widely used as a key material in the refrigeration and aerospace industries. A copper plate with a thickness of 0.05 mm and a diameter of 6 mm diameter was used as the arched sample to observe the frosting process on a cold surface. The arched sample was adhered to the arched sample holder with thermal-conductor adhesive tape (881, 3M, USA), and the holder was mounted onto the semiconductor unit with thermally conductive grease. During the test, the effect of the stress generated by the arched shape on the frosting process was ignored.

### 2.4. Experimental details

When the refrigeration unit was turned on, the surface temperature of the arched sample gradually decreased from the ambient temperature to the target temperature, and the frosting process was recorded during the temperature reduction process. The target temperature of the sample surface, which was -5°C, -10°C, -15°C, and -20°C, was obtained by adjusting the voltage across the semiconductor refrigeration unit. The ambient temperature and relative humidity of the experimental environment were kept constant at 18°C and 70%, respectively. During the test, the thickness of the frost layer on the cold surface was measured by the scaling method, where the calibration image was first taken and then the layer thickness was measured by counting the pixels in the experimental image against this scale. Each experiment was performed three times. In order to reduce experimental errors and random errors, the residual frost and water droplets on the sample surface were removed in an acetone ultrasonic bath for 3 min and in a deionized water ultrasonic bath for another 3 min. The samples were then dried in an oven at 60°C.

**Fig 3:**
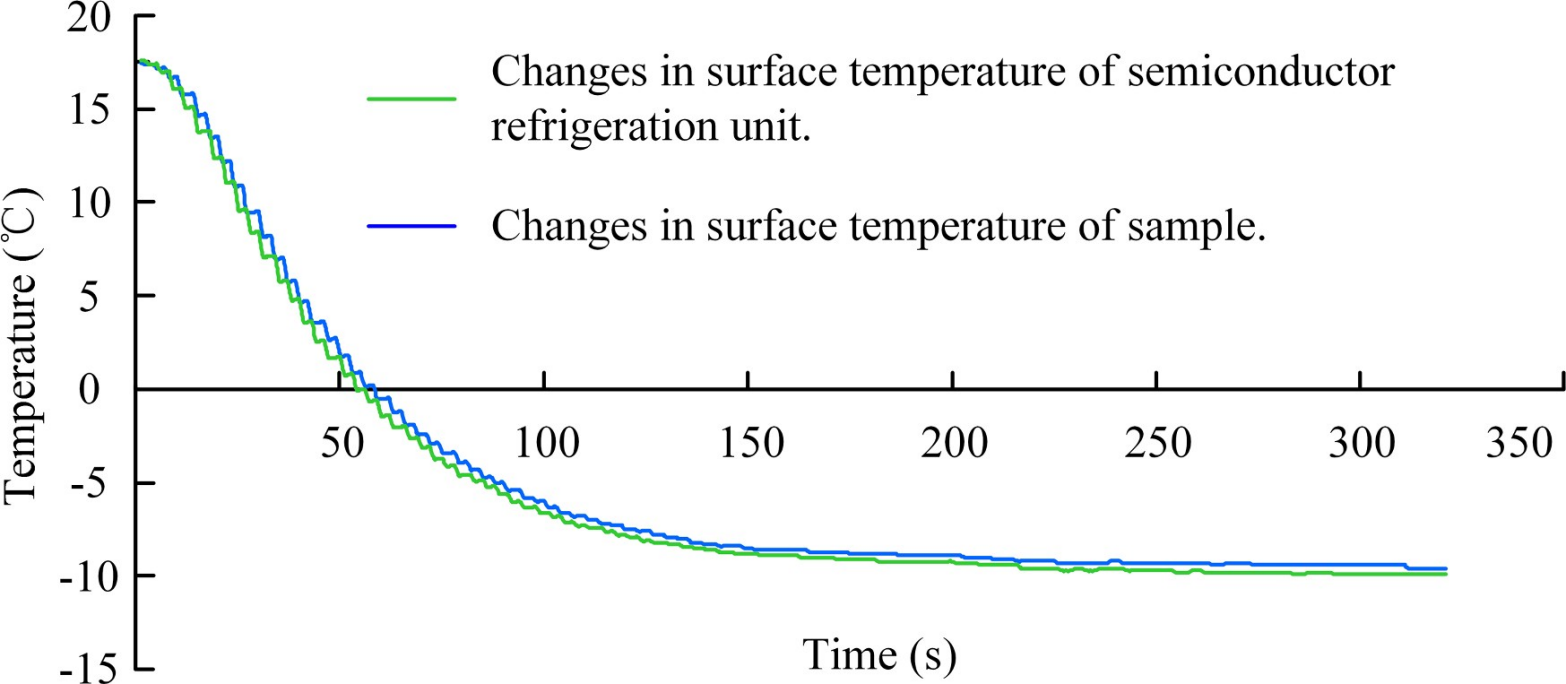
Temperature curves of cold surfaces.

To ensure that the sample surface was fixed at the set temperature to reduce the effect of the temperature difference between the sample surface and semiconductor refrigeration-unit surface on the frost-layer growth, both surfaces were measured with the K-type thermocouple. Hence, taking the target temperature of the cold surface at -10°C as an example, the temperatures of the respective surfaces of the sample and the semiconductor refrigeration unit were collected, and the results are shown in Fig 3. Owing to the small difference (approx. 0.3°C) between the surface temperatures of the sample and refrigeration unit, it can be concluded that the temperature difference had negligible effect on the frost-layer formation.

## 3. Results

### 3.1. Frost formation on arched sample

**Fig 4:**
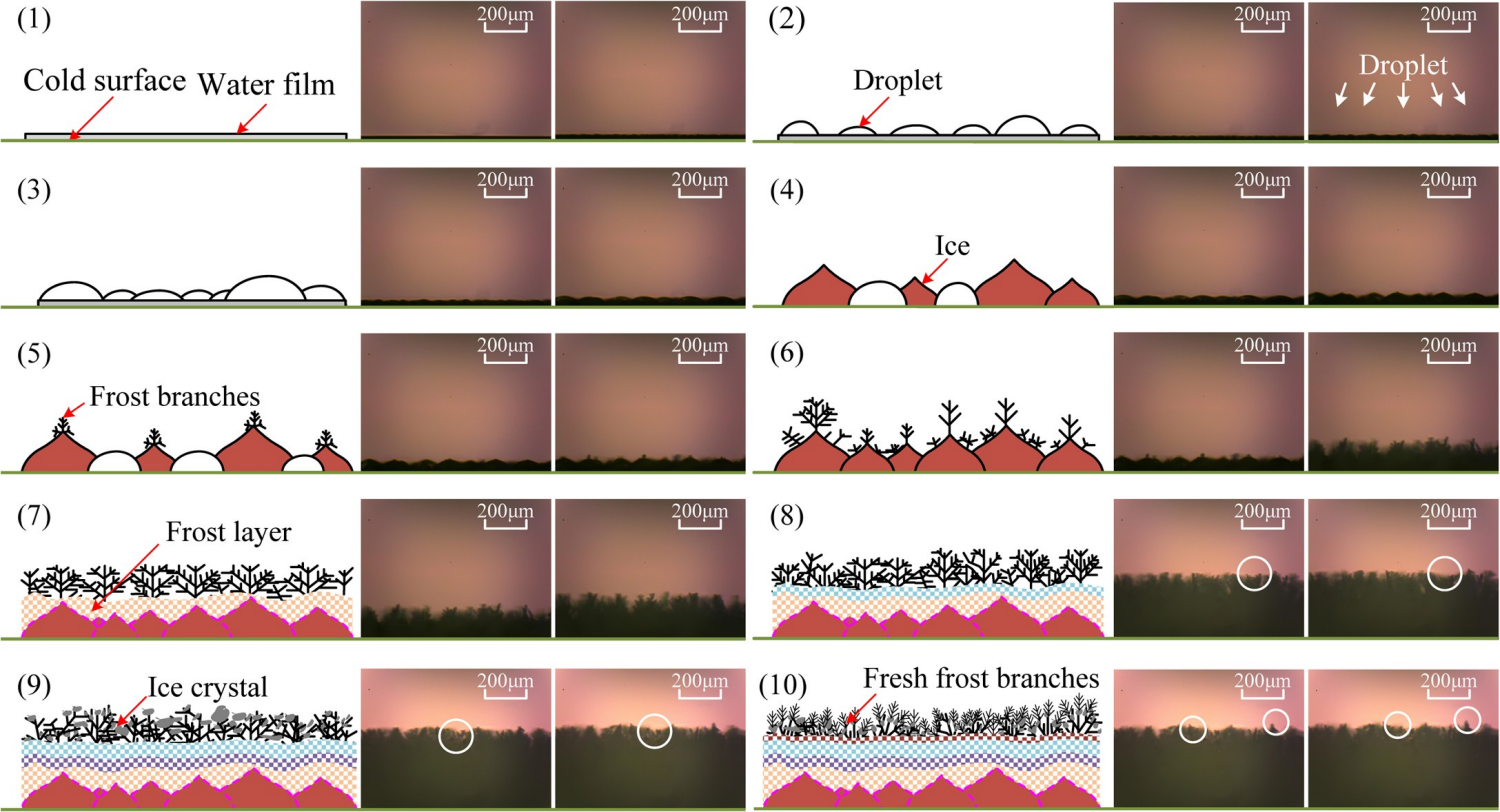
Schematic diagram and microscopic images of frosting process on surface of arched copper sample.

In order to optimize existing anti-frosting and defrosting methods and to develop new and more effective defrosting methods, it is necessary to understand the formation process of frost on a cold surface. Therefore, the frost-formation process on cold surfaces at different temperatures was observed during the test. For example, Fig 4 shows the frost-formation process on the cold surface of an arched sample at -15°C; the frost-formation process is also illustrated in a series of schematic diagrams. As apparent from this figure, the frosting process could be divided into two distinct stages: the ice-film-formation period and the frost-layer-formation period.

During the experiment, water vapor condensed on the cold arched-sample surface owing to the large difference between the surface temperature and the ambient temperature, and a water film gradually formed on the surface, as shown in Fig 4 (image 1). Many micro-droplets also formed (image 2 of Fig 4) simultaneously. When the surface temperature continued to decrease and remained above the freezing point, the micro-droplets began to converge into large droplets (image 3 of Fig 4). As cooling continued, a phase change started to take place and the droplets solidified into ice whose appearance was similar to that of a “peach” (image 4 of Fig 4). Next, the first frost branches appeared at the top of the peach shape (image 5 of Fig 4). Meanwhile, the frozen water film adhered to the arched sample through the built-up adhesion strength.

Next, new frost spots and frost branches appeared on the surface of the ice film, and the surface was gradually covered with frost branches (image 6 of Fig 4). During this period, frosting continued on the arched cold surface, which could be detected by observing the visibility on the microscope screen. The original frost branches grew rapidly and became thicker columnar frost branches. Many new and thin frost branches appeared on the main frost branches, and these frost branch started to grow in three dimensions. Moreover, the newly formed frost layer covered the old frost layer so that both the thickness and density of the frost layer gradually increased (images 7 to 10 in Fig 4).

However, owing to the gravitational force on the frost branches, air flow, and other factors, the bottom frost branches could not support the weight of the top of branches, and the bottom frost branches began to break or lodge. In addition, some frost branches outside the frost layer and the lodged frost branches began to melt, turning into ice crystals owing to the cold surface temperature. A new frosting process thus began. Hence, the entire frosting sequence on the arched sample could be separated into the appearance of first frost branches, rapid growth, frost-layer thickening, frost lodging, frost-branch melting, and the frost-crystal reformation.

### 3.2. Changes in frost branches

**Fig 5:**
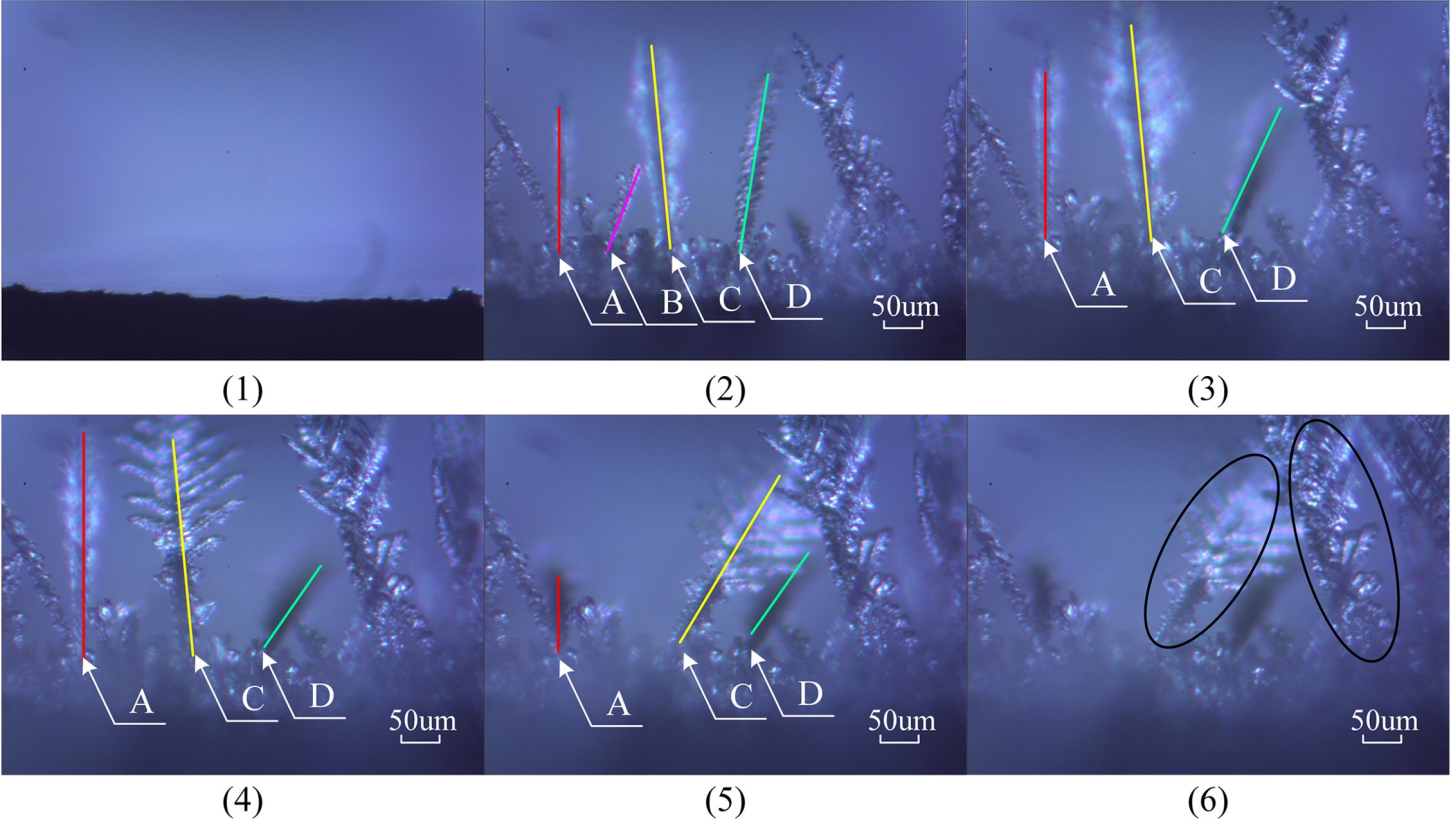
Changes of frost branches on the arched surface. (1) *t* = 0 s; (2) *t* = 74 s; (3) *t* = 84 s; (4) *t* = 94 s; (5) *t* = 97 s; (6) *t* = 104 s. The symbols A, B, C, and D mark the frost branches that changed during the frosting process.

Frost-branch breakage and lodging occurred during the observation test of the frosting process. Therefore, in order to observe the changes in the frost branches during the frosting process, the same device was used to observe in detail the changes in the frost branches on the arched copper sample’s surface under the same experimental conditions. Some of the captured images are shown in Fig 5.

The frost branches on the arched surface of the sample gradually grew, and the growth was accompanied by lodging, breaking, melting, and re-formation of frost crystals, as shown in Fig 5. The frost branches changed from one-dimensional growth in the vertical direction to three-dimensional growth in all directions. In the middle and top areas of the frost branches, additional new and tiny frost branches grew, and the complete cluster of frost branches became more and more sturdy.

The frost branch A gradually grew, then fell down onto the old frost layer (images 2–5 in Fig 5); the frost branch B melted after lodging (images 2 and 3 in Fig 5). The frost branch C grew into the main frost branch and formed many new frost branches, and its appearance changed from columnar to feathery. As shown in images 2–6 in Fig 5, frost branch C became tilted as it continued to grow.

### 3.3. Frost layer thickness on arched surface

**Fig 6:**
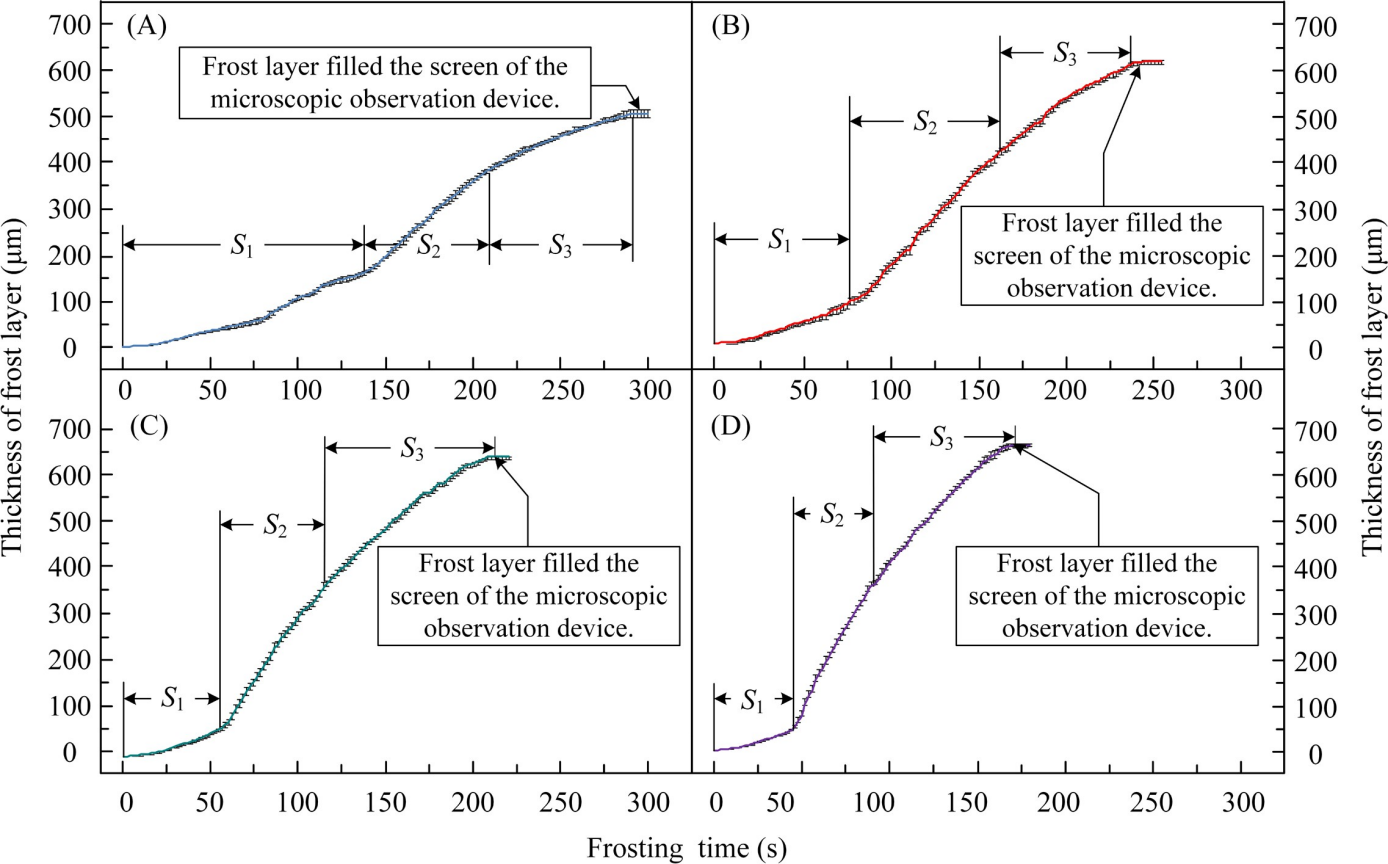
Changes in thickness of frost layer on the surface at different temperatures. (A) Surface temperature: -5°C; (B) surface temperature: -10°C; (C) surface temperature: -15°C; (D) surface temperature: -20°C. *S*_1_, *S*_2_, and *S*_3_, represent the durations of different stages during the frosting process.

The thickness of the frost layer on a cold surface at different temperatures was measured by setting the scale, and the measured results are shown in Fig 6. During the experiments, the frost layer was defined to have reached its maximum thickness when it filled the entire viewing screen of the microscopic observation device. As the surface temperature of the arched sample decreased, the thickness of the frost layer on the surface gradually increased, and the time required for the frost layer thickness to reach its maximum became shorter. For example, the frost layer on the surface of the arched sample at -20°C could reach a maximum thickness of 660 μm during the experiment, which is approximately 160 μm higher than the maximum frost-layer thickness on the surface of the sample at -5°C.

When the surface temperature of the sample (*T*_S_) was lower, the time of frost-branch appearance on top of the ice was shorter, i.e., △*T*_S1A_>△*T*_S1B_>△*T*_S1C_>△*T*_S1D_, and the thickness of the water film on the sample surface during the period S_1_ was thinner, i.e., *L*_S1A_>*L*_S1B_>*L*_S1C_>*L*_S1D_, as shown in Fig 6. For instance, compared with the change in frost layer thickness on the surface at -10°C, the condensation period of water film was much longer when the surface temperature was -5°C, namely almost half of the total recording time, i.e., ~135 s, and the water film reached the maximum thickness of 194 μm during four experiments at different sample surface temperatures.

The formation rate of the frost layer rapidly increased with decreasing surface temperature. The frost layer had the lowest growth rate on the sample surface at -5°C during the four different tests. Moreover, the growth rate of the frost-layer thickness gradually decreased as the cooling continued. For example, the growth rate during the period *S*_2_ was lower than the rate during the period *S*_3_.

According to the variations in thickness of the frost layer, the complete frost-formation process on the arched copper sample surface could be summarized as the ice-film formation period (*S*_1_), the rapid-growth stage of the frost layer (*S*_2_), and the stable-growth stage of the frost layer (*S*_3_). In other words, the rapid-growth stage included the frost-branch appearance and rapid growth, while the stable-growth stage consisted of frost-layer thickening, frost lodging, frost-branch melting, and frost-crystal reformation.

## 4. Discussion

As observed during the experiment, water vapor first condensed on the arched surface and then solidified into ice. This means there was an ice layer before the frost formation, and the ice adhesion strength gradually build-up. This was unfavorable to thorough clearance of the frost layer during the actual defrosting process. Hence, if the hydrophobicity of the substrate surface was improved by adopting surface modification technologies, the water content would be decreased and the formation of ice would be delayed. Meanwhile, the hemispherical shape of the frozen droplets provided more nucleating points for the growth of the frosting branches.

**Fig 7:**
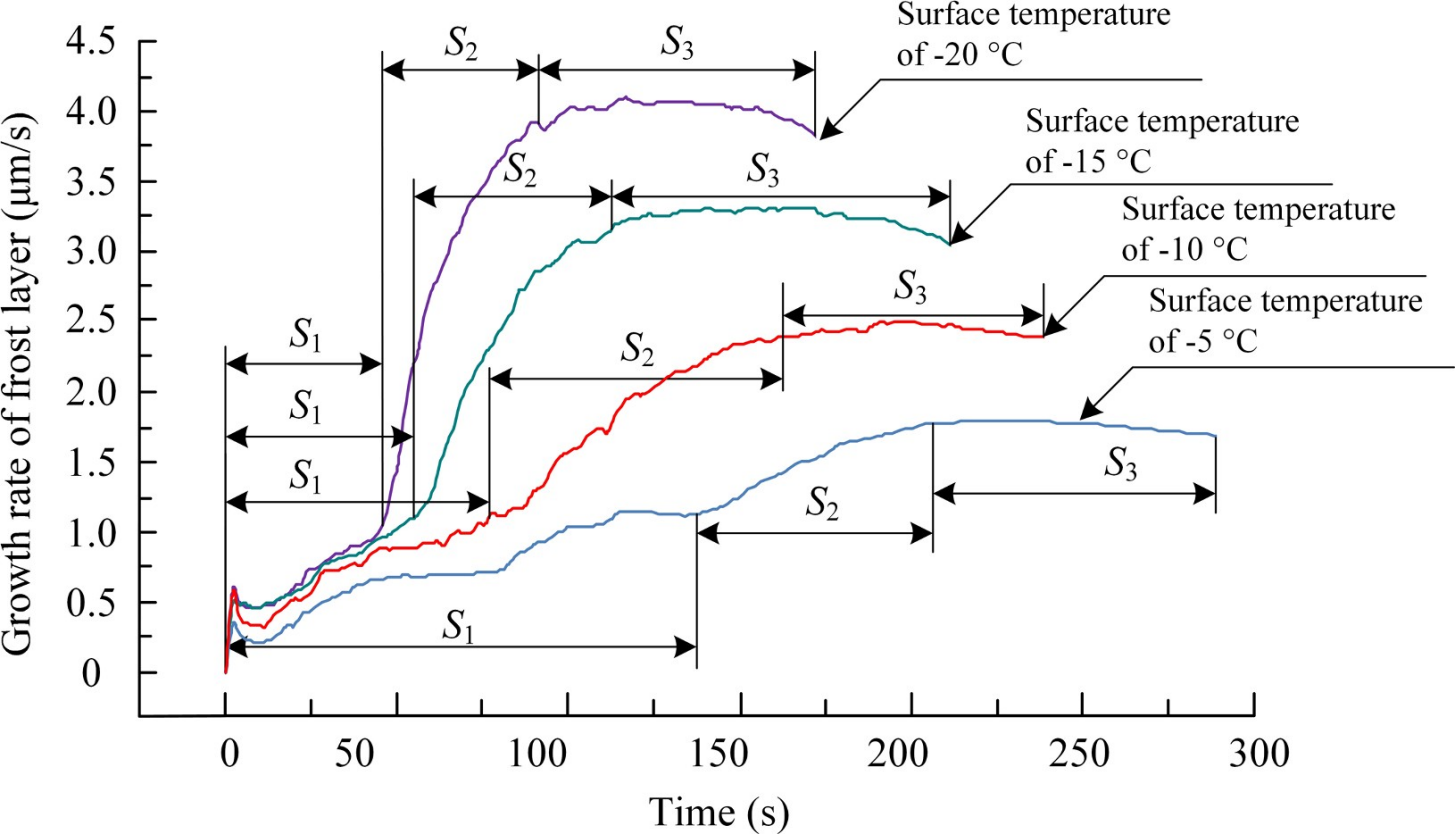
Growth rate of frost layer on the arched surface. *S*_1_, *S*_2_, and *S*_3_, represent the durations of different stages during the frosting process.

In order to better understand the growth characteristics of the frost layer, its growth rate on the arched surface at different surface temperatures was calculated, as shown in Fig 7. Combining Figs 4 to 7, it can be seen that during the rapid-growth stage, frost branches developed on the ice film mainly in a columnar shape and grew rapidly along the vertical direction of the sample surface (as shown in the Fig 4, and again by the *S*_2_ interval in Fig 7). The thickness of the frost layer increased rapidly, as shown by the *S*_2_ interval in Fig 6. However, the rapid growth of frost branches resulted in large gaps (Fig 5) between adjacent frost branches, resulting in a rapid increase in the thickness of the frost layer. The frost layer produced during the rapid growth of the frost branches was loose and had low density. Moreover, the loose frost layer and the undulating surface of the ice film would increase the probability of lodging or breakage of frost branches, as well as melting and re-frosting in the next period. If there was a way to disturb the rapid growth of the frost layer, the formation of the frost layer could be prevented.

**Fig 8:**
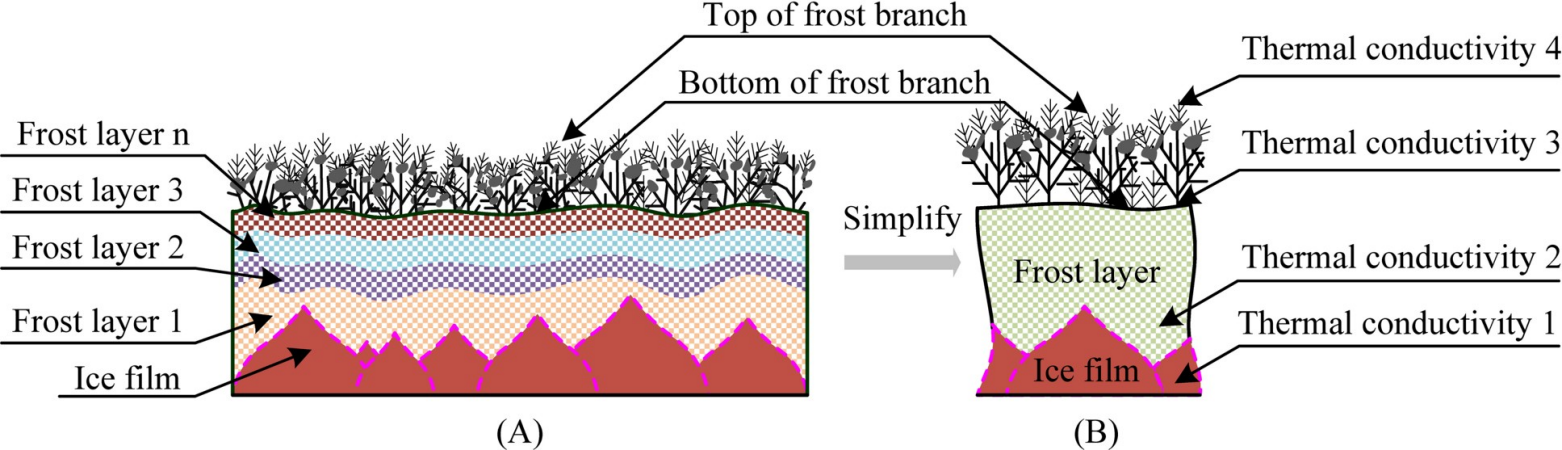
Structure and thermal conductivity model of the frost layer. (A) Composition of frost layer; (B) thermal conductivity model of frost layer.

According to the frost-formation process, the ice film on the sample surface, the multiple frost layers, and the frost branches constitute the frost layer covering the surface, thus yielding a frost layer with different properties such as density and thermal conductivity, as shown in Fig 8. During the stable growth of the frosting process, the newly formed frost layer covered the original frost layer, and the frost layer became denser, which could be directly seen as decreasing visibility of the frost layer on the observation device. Although the frost layer was dense during the stable-growth period, the surface of the frost layer was still soft compared to the growth of the rigid ice-film surface, which could not provide more space for the growth of the frost layer.

Owing to the different thermal conductivities within the frost layer, there was a temperature difference between the top of the frost branches and the substrate surface, but the temperature at the top of the frost branches was still below the freezing point of water. Meanwhile, the top of the frost branches on the surface was surrounded by air at 18°C, and the large temperature difference between the top of the frost branches and the environment caused the top of the frost branches to melt. This increased the load of the frost branches and the possibility of lodging. Therefore, the different thermal conductivities decreased the growth rate of the frost layer.

Meanwhile, as the frost branches continuously grew in three dimensions, water vapor adhered to the frost branches and promoted the formation of more new frost branches. Owing to the frost branches adhering to the undulating surface of the ice film, the increasing weight of the upper frost branches could cause the entire cluster of frost branches to tilt, lodge and break, as shown in Fig 5. The lodged and broken frost branches were buried in the existing frost layer and became the nucleating sites for many tiny frost branches. The phenomenon of frost-branch breaking, lodging and melting reduced the growth rate of the frost layer, and the thickness of the frost layer slowly increased, as shown in Figs 6 and 7. However, the density of the frost layer increased and the frost layer became tighter during this period, and conventional defrosting methods could be used to remove the accumulated frost.

## 5. Conclusion

In order to observe the true frosting process on material’s surface and mitigate the edge effect on observation of the frosting process, a specially built apparatus was used to observe the frosting process on the arched surface of a copper sample at different surface temperatures of -5°C, -10°C, -15°C, and -20°C. The characteristics of the frost branches and frost layer during the frosting process were analyzed.

The experiments showed that the frosting process on the arched surface could be separated into the ice-film-formation period, rapid-growth period of the frost layer, and stable-growth period of the frost layer. In addition, the surface temperature significantly affected the frosting characteristics, such as the thickness and growth rate of the frost layer, during the frosting process. When the surface temperature was lower, the longer the water vapor froze into the ice film, the faster the growth of the frost layer, and the thicker the frost layer formed on the material surface during the frosting process. Compared to the other three tests with different surface temperatures, an ice film formed on the sample surface at -20°C after 42 s, and it reached a maximum growth rate of 4.14 μm/s, yielding a frost layer with the highest thickness of about 660 μm.

Furthermore, we considered that the frost layer on the material surface consisted of many newly formed frost layers and ice film, which all had different properties at different heights. Hence, the frost branches were prone to breaking, lodging, and melting owing to the undulating surface of ice film, the frost layer with different thermal conductivities, and the top-heavy feathery frost branches. The changes in the frost branches and the soft surface of the frost layer could not provide more nucleating points for the frost layer growth or support the growth space, thus limiting or affecting the growth and formation of the frost layer.

In conclusion, the results of the experiments demonstrate the detailed frosting process on the arched surface. Based on the observed phenomena during the experiment, conventional defrosting methods could be used to disturb the frosting process and attain surface frost removal. Some of methods include preventing or delaying the formation of ice film, slowing the growth rate of the frost layer, making the frost branches lodge, break, and melt.

## Supporting information

S1 Dataset The thickness of frost layer on the surface at different temperatures. (XLSX)
